# Moving in time: Bayesian causal inference explains movement coordination to auditory beats

**DOI:** 10.1098/rspb.2014.0751

**Published:** 2014-07-07

**Authors:** Mark T. Elliott, Alan M. Wing, Andrew E. Welchman

**Affiliations:** 1 School of Psychology, University of Birmingham, Edgbaston B15 2TT, UK; 2 Department of Psychology, University of Cambridge, Cambridge CB2 3EB, UK

**Keywords:** movement synchronization, Bayesian inference, sensory integration, motor timing

## Abstract

Many everyday skilled actions depend on moving in time with signals that are embedded in complex auditory streams (e.g. musical performance, dancing or simply holding a conversation). Such behaviour is apparently effortless; however, it is not known how humans combine auditory signals to support movement production and coordination. Here, we test how participants synchronize their movements when there are potentially conflicting auditory targets to guide their actions. Participants tapped their fingers in time with two simultaneously presented metronomes of equal tempo, but differing in phase and temporal regularity. Synchronization therefore depended on integrating the two timing cues into a single-event estimate or treating the cues as independent and thereby selecting one signal over the other. We show that a Bayesian inference process explains the situations in which participants choose to integrate or separate signals, and predicts motor timing errors. Simulations of this causal inference process demonstrate that this model provides a better description of the data than other plausible models. Our findings suggest that humans exploit a Bayesian inference process to control movement timing in situations where the origin of auditory signals needs to be resolved.

## 1. Introduction

Many human activities, from holding a conversation to playing music, have their basis in our ability to extract meaningful temporal structure from incoming sounds. For rhythmical structures in particular, humans identify key events and extract the underlying ‘beat’ of the auditory signals [1]. Such auditory rhythms promote movements ‘in time’ with the beat with little apparent effort [2,3], as demonstrated through the capacity to dance or play along to music comprising multiple rhythmic streams. Yet, for such complex stimuli, it is unknown how temporal events are extracted and chosen as the targets to which movements are synchronized.

The complexity of incoming auditory signals is partially reduced by early sensory processing that filters out irrelevant auditory information [4]. Nevertheless, auditory signals of interest may still consist of multiple components. For instance, keeping in time with other players in a quartet involves sensing different sequences of tones (e.g. the notes played on the viola versus cello) that share an underlying rhythm but are likely to fluctuate in relative phase, depending on how well each player can remain in time with the rest of the group [5]. Based on these discrepancies, the brain must determine whether to integrate relevant components into a single stream or treat them as separate [6].

In multisensory settings, the decision to integrate cues or treat them as separate sources is well captured using the Bayesian framework of causal inference [7–10], based on the statistical probability that sensory events relate to a single event in the environment versus multiple events. If there is evidence that sensations originate from a single environmental cause, the sensory cues are combined in a statistically optimal way across modalities to gain the best estimate of an object or event [11–13]. Within a single modality, there is also evidence for statistically optimal combination, for instance in combining distinct visual features such as disparity, motion or texture [14–16]. Critically, however, such integration is believed to be mandatory. Here, we test for the integration of within-modality auditory cues to time. We evaluate whether the brain applies a causal inference process to determine the circumstances under which auditory sequences (distinguished only by tone frequency) should be integrated into a coherent estimate of rhythm or separated into distinct events.

First, we develop a Bayesian causal inference model for movement synchronization that describes the scenarios under which a regular stream of sensory cues from same-modality sources are integrated. Then, we test the model by asking participants to tap their index finger in time to auditory sequences that comprised two auditory metronomes presented simultaneously with equal mean tempo. To test the causal inference process, we manipulated these cues in two different ways. First, we applied a phase offset between the metronomes, such that the beats from one metronome occurred shortly before the other. Second, we varied the temporal reliability of the metronomes such that rather than having isochronous beat onsets, they varied randomly around the (underlying) isochronous onset time (referred to as ‘jitter’). By adding small levels of jitter to one metronome and large levels of jitter to the other, we manipulated the relative reliability of the two metronome sources [17]. Thus, we were able to observe changes in the timing and variability of participants' finger taps and assess the conditions under which the cues appeared to be integrated or treated as separate. Finally, we test four models and fit the simulated results to the experimental data to investigate whether causal inference best describes the observed results. We found that a causal inference model that adjusts for a consistent phase offset between cues demonstrated a fit close to the empirical data, exceeding that of alternative models based on the exclusive integration or exclusive separation of cues.

## 2. Material and methods

### (a) Participants

Staff and students from the University of Birmingham were recruited to participate in the experiment. Participants provided informed consent and were screened for sensory and motor deficits. Nine participants (seven male, four left-handed, mean age: 29.8 ± 5.9 years) took part. Five participants had some musical expertise (i.e. currently play a musical instrument; mean years of experience = 10.8).

### (b) Experimental set-up

Participants sat at a table wearing a pair of headphones (Sennheiser EH150) through which the auditory metronome cues were presented. They tapped the index finger of their dominant hand in time to the metronome on to a wooden surface mounted on a force sensor. Responses were registered using a data acquisition device (USB-6229, National Instruments Inc., USA). Metronome presentation was controlled using the MatTAP toolbox [18].

### (c) Metronome stimuli

**Figure 1.**
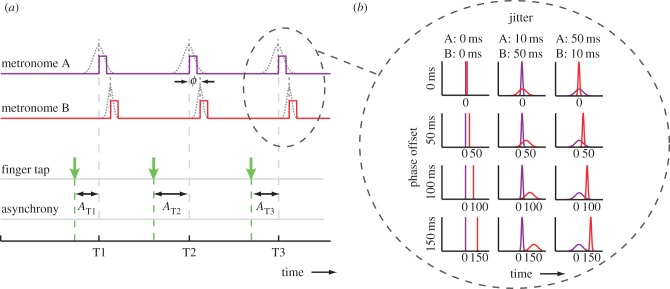
(*a*) An illustration showing the timing relationships between the two metronomes and the calculation of asynchronies. The square pulses show the regular onset time of the metronome beats (before jitter applied). Both metronomes had the same underlying period, and metronome B had a phase-offset delay from metronome A of *ϕ* = 0, 50, 100 or 150 ms. To create temporal uncertainty, a random perturbation (‘jitter’) was added to the regular onset time of each beat. The s.d. of the jitter distribution differed between metronomes (A, B: {0, 0 ms}; {10, 50 ms}; {50, 10 ms} (depicted)). Asynchronies (*A*) were calculated between the finger-tap onsets and the onset of metronome A, before jitter was applied. (*b*) Probability distributions of metronome beat onsets. Illustration showing the expected distributions of beat onsets relative to the regular beat onset of metronome A (0 ms). Distributions are shown for each phase offset (row) and jitter condition (column). (Online version in colour.)

The auditory stimuli consisted of two independently controlled auditory metronomes (metronome A, pitch 700 Hz; and metronome B, pitch 1400 Hz; figure 1*a*). The metronomes were offset in phase (0, 50, 100 or 150 ms), such that metronome B followed metronome A (pilot testing established that pitch order (low–high versus high–low) did not influence synchronization behaviour). Metronome beats lasted 30 ms and the period was varied randomly (by the same amount for A and B) across trials with a value between 470 and 530 ms, to minimize learning of tempo across trials and encourage adaptive correction within trials.

To manipulate metronome reliability, we applied temporal jitter independently to each metronome (figure 1*b*) by perturbing the regular onset of the metronome beat by a random value sampled from a Gaussian distribution (*μ* = 0; *σ* = 10 or 50 ms). We tested the effect of differing reliabilities between the two metronomes and whether this would influence finger movement onsets and variability. Hence, we used the following jitter conditions (s.d. for metronome A and metronome B): {10, 50 ms}, {50, 10 ms} and a baseline condition where both metronomes were reliable {0, 0 ms}.

Participants were not explicitly informed that the auditory cues consisted of two metronomes. Instead, they were instructed to ‘tap in time to the metronome’ with some trials appearing ‘harder to tap along to than others’. This instruction was intended to encourage participants to use both cues and not attempt to ignore one in favour of the other.

Participants completed 10 trials per condition (12 conditions in all), with each trial consisting of 30 metronome beats. Conditions were randomized across trials to minimize any prior expectation about the metronome statistics building up across trials. To allow participants to build up prior knowledge of the metronome within a trial, analyses were performed on the tap-metronome asynchronies of beats 15–28 (the last two were ignored to discount anticipation or termination effects at the trial end [19]).

To determine baseline movement synchronization behaviour, we also presented 30 trials on which a single metronome was presented, where the degree of jitter applied was varied across trials (0, 10 or 50 ms).

### (d) Analysis

To quantify synchronization behaviour, we measured the time difference between the onset of the participants' finger taps and the metronome beat (asynchrony; figure 1*a*). We referenced all metronome beats relative to the onset of metronome A (prior to any jitter perturbations) to provide a consistent, static reference point for all trials regardless of condition. Negative asynchronies indicated that the finger tap preceded the onset of the beat. We calculated the s.d. of asynchronies within a trial across participants and conditions. A repeated measures ANOVA was used to determine any significant effects of phase offset or jitter on the asynchrony s.d. We quantified the distribution of asynchronies for each participant, grouped by condition and tested the experimental data for unimodality or bimodality using Gaussian mixture models (GMMs) with either one or two centres. Mean asynchronies were then calculated based on the best fitting GMM distribution.

For comparison with the simulated asynchrony distributions of the models we tested, we fit the empirical data with probability density functions (PDFs). These were estimated using a Gaussian kernel density estimator (KDE) [20] method implemented in Matlab [21].

### (e) Sensorimotor synchronization based on a causal inference model

**Figure 2.**
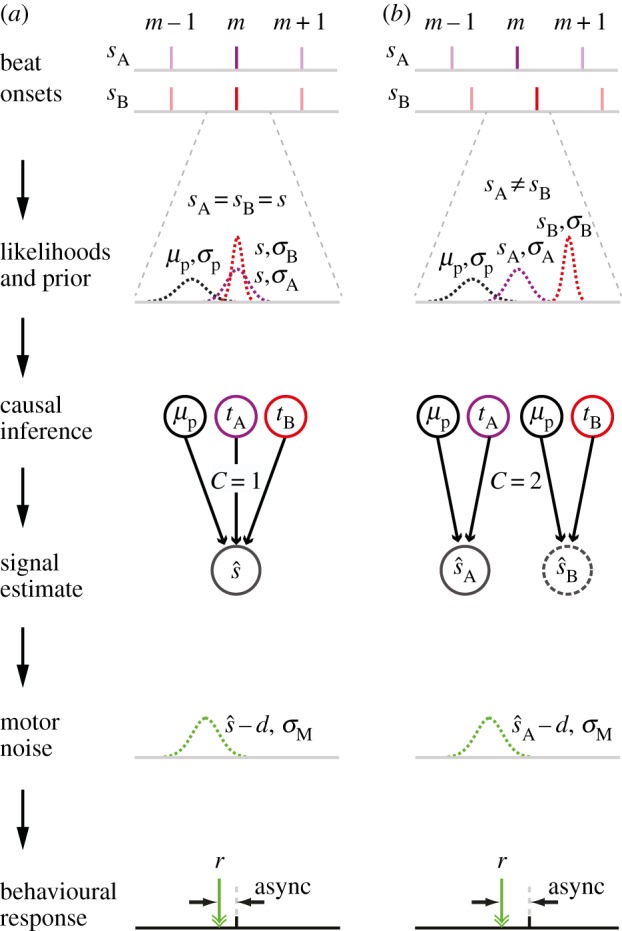
Schematic of the causal chain. (*a*) Common stream: signals are generated from a common source (*s*_A_ = *s*_B_ = *s*). The sensory likelihood distributions for the two metronome signals are modelled by Gaussian distributions *N*(*s*, *σ*_A_) and *N*(*s*, *σ*_B_), respectively, where *σ*_A_, *σ*_B_ describes the uncertainty in the sensory registration. The observer has an expectation of where the *m*th beat should occur, centred around a time *μ*_p_ relative to the true beat *s*. This prior expectation is equal to the difference between their beat onset estimate *ŝ* and the true onset time *s* on the preceding (*m* − 1th) beat and is modelled as a Gaussian distribution *N*(*μ*_p_, *σ*_p_), where *σ*_p_ defines the strength of the prior. The observer estimates the cue onset times *t*_A_ and *t*_B_, which are sampled from the likelihood distributions. Using the information from *μ*_p_, *t*_A_ and *t*_B_, the causal inference process results in evidence that the signals define a common beat (*C* = 1), and the estimated signal onset time *ŝ* is calculated as a weighted average of *μ*_p_, *t*_A_ and *t*_B_. The reliability of the three distributions 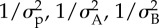 defines the relative weightings. The observer plans their movement to coincide with the estimated beat *ŝ*, introducing motor noise *σ*_M_, and an anticipation effect *d*, which results in the observable asynchrony between the movement *r*, and the true beat *s*. (*b*) Independent streams: two signals are generated from independent sources (*s*_A_ and *s*_B_). The sensory estimation process is the same as for (*a*); however, the prior is defined as the difference between their beat onset estimate *ŝ* and the true onset time *s*_A_ on the *m* − 1th beat. Based on *μ*_p_, *t*_A_ and *t*_B_, the causal inference model has more evidence that signals are independent (*C* = 2). Signal onset estimates *ŝ*_A_ and *ŝ*_B_ are therefore calculated independently as the weighted average of *μ*_p_, *t*_A_ and *μ*_p_, *t*_B_, respectively. Similarly, the relative weightings are proportional to their reliabilities, 
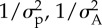
 and 
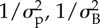
. As the observer has two estimated signal onsets, they select one with which to synchronize their movement. That is, the observer will define the signal onset estimate to be either *ŝ* = *ŝ*_A_ (as depicted in figure), or *ŝ* = *ŝ*_B_. This choice varies for each beat, with observers' esoteric preferences dominating the relative reliability of the two signals. The observer plans their movement to coincide with the estimated beat *ŝ*, introducing motor noise *σ*_M_, and an anticipation effect *d*. This results in the observable asynchrony between the movement *r*, and the referenced true beat (always *s*_A_, to match experimental analyses). (Online version in colour.)

Here, we outline the features of the simulated task where an observer uses Bayesian causal inference to synchronize their movements. An overview is shown schematically in figure 2 while the full model derivation is provided in the electronic supplementary material, A.

The observer's task is to synchronize their movements to rhythmic auditory cues presented to them. The cues consist of two discrete tones of different pitch (*s*_A_ and *s*_B_). The observer must estimate the onsets of the underlying beats produced by the auditory cues to make movements in synchrony with those beats. They do this using a causal inference process based on: (i) the likelihood of the onsets of the two auditory cues, whose true onset times are corrupted by sensory noise; and (ii) the prior expectation of where the beat will occur, which is based on the previous beat onset estimate [22,23]. The causal inference process allows the observer to determine whether the two auditory cues should form a single common beat and hence combine the likelihood of the two beats with the prior to obtain the estimated onset time of that beat (*ŝ*; figure 2*a*). Alternatively, if the causal inference process indicates that the two auditory cues are in fact independent, then two beat onset times are estimated (*ŝ*_A_, *ŝ*_B_) based on the prior and likelihood of each independent cue onset (figure 2*b*). In this latter scenario, the observer must choose one of the estimated beat onsets as the target for movement synchronization. Here, we assume the observer has a bias in selecting one stream over the other (regardless of the cue statistics). Based on this bias, the observer will select auditory cues from stream A on a certain proportion of trials, and stream B for the complementary remainder of trials. The beat from the selected stream defines the single beat onset estimate (*ŝ*). Finally, the observer produces a motor action which is aligned with the estimated beat onset time, but subjected to a negative motor delay (representing the anticipation effect observed in many sensorimotor synchronization studies see [24]) and motor noise. The resulting output we observe is an asynchrony between the observer's movement and the ‘actual’ beat which, to be consistent with the experimental analyses, we take to be the true onset time of auditory cue *s*_A_.

### (f) Model comparisons

We compared three alternative models to the causal inference model described above (denoted CI), to see which best described the experimental data. First, we tested a model of mandatory integration (MI), where the observer always considers the two cues to form a single common beat, regardless of the cue statistics. Similarly, we considered a mandatory separation (MS) model, where the observer always deems the cues independent and estimates the onset for two corresponding independent beats (subsequently selecting the preferred beat for movement synchronization). Finally, we tested an alternative causal inference model that included ‘phase-offset adaptation’ (CI_PA_), where any *consistent* phase offset between cue A and B across beats is accounted for in the inference process and hence is disregarded in the judgement of whether the cues form a single beat or independent beats. We tested this extra model to see whether the fixed phase offsets we applied in the experimental conditions resulted in participants adjusting their judgement of the level of deviation required between cues before they were considered separate beats.

### (g) Parameter fitting to participant data

We developed simulations to test whether the models detailed above could describe the experimental results. For each model, we generated 2000 simulated finger-tap asynchronies for an observer synchronizing to auditory rhythmic cues that matched the statistics of the experimental phase offset and jitter conditions. The simulated asynchronies for each condition were converted into likelihood functions for the model using an optimized Gaussian KDE [20]. Three free parameters were used to fit each participant's asynchrony data to the model output likelihood function for each condition (see the electronic supplementary material, A): the strength of the prior expectation of the time of the next beat (*σ*_p_; range (10, 500) ms); the prior probability that the two cues will form a common single beat (*p*_single_; range (0, 1), fixed to 0 for the MS model and 1 for the MI model) and the negative asynchrony offset (*d*; range (−100, 100) ms). A fourth free parameter, *β* was fitted to experimental data from a single condition (phase offset: 150 ms, jitter: {0, 0 ms}) to describe the proportion of time the observer shows preference to cues from stream A versus stream B. This parameter was applied to the remaining conditions. We used a global search algorithm [25] that sequentially interchanged data between four different meta-heuristic optimizers (genetic algorithm, particle swarm optimization, differential evolution and simulated annealing) to ensure robust parameter optimization. The fitting algorithm minimized the negative log-likelihood of each participant's data for each simulated condition.

To test the relative fit of the models to the data, we used the Bayesian information criterion (BIC; [26]). This measure shows the log-likelihood of the data given the model and penalizes for the number of free parameters. BIC scores were summed across conditions for each participant. The differences in BIC scores between models were calculated and averaged across participants.

Similarly, we calculated the goodness of fit using the BIC. To get an overall equivalent *r^2^* value, BIC values (*L*(*θ*)) were compared to two points of reference [27]. The first point of reference was the BIC of the data to a probability distribution of the data itself (*L*(Max); fitted using a Gaussian KDE)—i.e. the best fit achievable. The second was the BIC of the data to a random distribution (*L*(Rand); fitted using a cubic spline)—i.e. giving a worst case fit. The goodness of fit *r^2^* was scaled to a value between 0 and 1 as follows:2.1
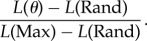


## 3. Results

### (a) Experimental results

To test the role of a causal inference process when synchronizing movements to multiple streams of auditory events, we asked participants to tap their index finger in time with beats defined by two metronomes (A and B) that could differ in their temporal reliability (jitter) and their phase offset (B relative to A). We measured the time difference (asynchrony) between the onsets of metronome A and the corresponding finger taps. A standard approach to quantifying synchrony performance is to calculate the variability (s.d.) of asynchronies across conditions [28]. Here, we initially use that approach to identify the effect of the jitter and phase-offset conditions on participants' performance. Subsequently, we apply more detailed analyses on the asynchrony distributions.

We expected that when tapping to single metronome beats, the asynchrony s.d. would increase with increasing jitter applied to the metronome. By contrast, when two metronome streams were presented in parallel, one with high jitter, the other with low jitter, we predicted that participants would integrate the cues and the resulting asynchrony s.d. would remain low [17,29]. We further predicted that this integration effect would reduce with increasing phase offset, as participants become more ready to treat the cues as originating from independent beats. Under this scenario, we expected the asynchrony s.d. to increase with increasing phase offset between metronomes.

**Figure 3.**
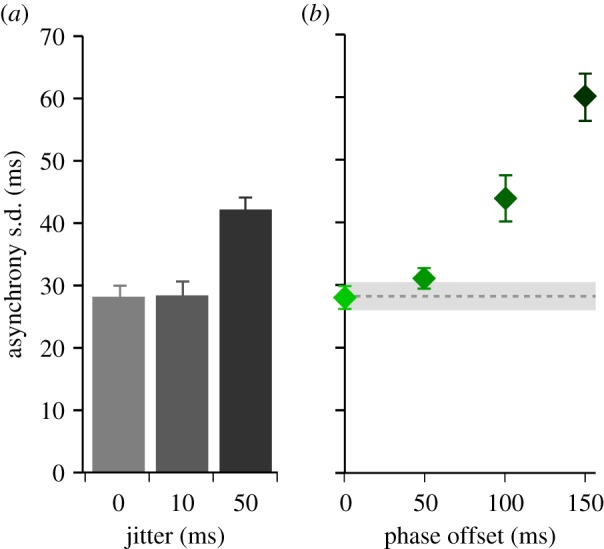
(*a*) Asynchrony s.d. to single metronome presentations. The metronome was jittered by 0, 10 or 50 ms. Error bars show s.e.m. (*b*) Asynchrony s.d. in the dual metronome conditions as a function of phase offset and averaged across jitter conditions. Error bars show s.e.m. The horizontal grey bar indicates the asynchrony s.d. in the isochronous single metronome condition, ±1 s.e.m. (Online version in colour.)

First, we manipulated metronome jitter to verify that asynchrony variability was affected, presenting a single metronome jittered by 0, 10 or 50 ms. We measured the asynchrony variability (s.d.) of the finger taps relative to the underlying unjittered metronome beats. As expected, increasing the jitter resulted in higher asynchrony variability (*F*_2,16_ = 37.6, *p* < 0.001; figure 3*a*).

Next, we considered synchronization performance when two metronomes (A and B) were simultaneously presented, one with high levels of jitter (50 ms) and the other only slightly jittered (10 ms). In particular, we focused on the zero phase-offset conditions and compared the asynchrony s.d. (averaged over the two jitter conditions: {10, 50 ms} and {50, 10 ms}) to that observed in the unjittered single metronome condition. Using a paired *t*-test, we found no significant difference between these two conditions (*t*_8_ = −0.71, *p* = 0.497). Hence, in contrast to the single metronome conditions where jitter substantially impacted on participants' performance, asynchrony variability remained low in the dual metronome condition, even though one of the metronomes was highly jittered. We further found no main effect of jitter on asynchrony s.d. (*F*_1.2,9.5_ = 1.5, *p* = 0.255) when we analysed all conditions for the dual metronome presentations. This means that asynchrony variability did not increase regardless of whether one of the metronomes was highly jittered or both were isochronous. These results highlight that participants were able to take advantage of the more reliable metronome to maintain their synchronization performance in the dual metronome conditions.

We found that, as predicted, an increasing phase offset between the two metronomes increased the asynchrony s.d. (*F*_1.6,12.8_ = 27.1, *p* < 0.001; figure 3*b*). While there was no difference between 0 and 50 ms phase offsets (*p* = 0.311), asynchrony s.d. increased significantly at phase offsets of 100 ms (*p* = 0.042) and 150 ms (*p* = 0.001). We suggest that these results indicate two different strategies in participants' synchronization. At low phase offsets, participants are integrating the two cues into a single beat estimate, with the outcome that the asynchrony s.d. remains low. However, as the phase offset increases, participants are treating the cues independently and switching between them. This switching incurs a substantial increase in asynchrony variability, regardless of the jitter applied to each metronome.

**Figure 4.**
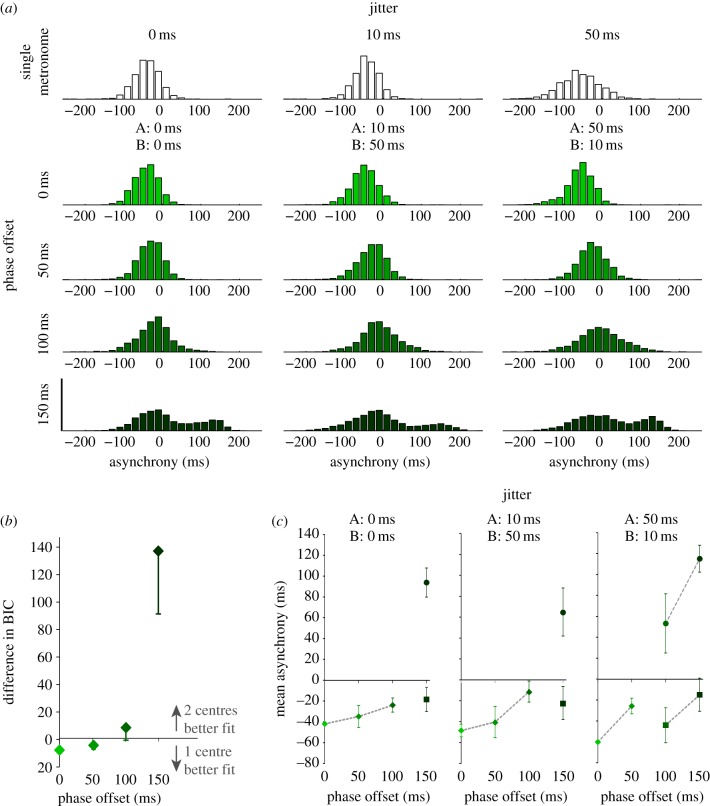
(*a*) Histograms of asynchronies from the experimental data. Negative asynchronies indicate the tap preceded the onset of metronome A. Histograms are shown for each phase-offset condition (rows). Each column plots histograms for the different jitter conditions: {0, 0 ms}, {10, 50 ms} and {50, 10 ms}. (*b*) Difference in Bayesian BIC values for GMM fits as function of phase offset. GMMs were fitted to each participant's data with either one or two centres. The goodness of fit of the data to each of the GMMs was measured using the BIC. The difference in BIC was calculated across conditions for each participant and averaged to determine whether the histogram of data was more likely to originate from one or two distributions. Negative values indicate a better fit to the single centred GMM; positive values indicate a better fit to the two centred GMMs. Error bars show s.e.m. (*c*) Mean asynchronies based on GMM centres. Mean asynchronies were calculated based on the GMM centres that fitted best to each participant's data. The figure shows the mean asynchronies for unimodal distributions (diamond symbols) except where more than half the participants demonstrated better fits to bimodal distributions. Where this occurs, we plot the mean of both centres (lower value, squares; higher value, circles). Plots are shown for each jitter condition. Error bars show s.e.m. (Online version in colour.)

To examine these apparent strategies in more detail, we considered the distributions of asynchronies in each condition. Visual inspection indicated unimodal distributions at low phase offsets (suggesting integration of cues) and bimodal distributions at larger offsets (suggesting independent targeting of the cues) (figure 4*a*). We quantified this observation by fitting two GMMs to each participant's data: either with one centre (indicating a unimodal distribution) or two centres (indicating a bimodal distribution). The difference between the BIC values for the two GMM models was calculated to establish which provided a better fit. We found that at low phase offsets (0, 50 ms), a unimodal distribution was more likely, while bimodal distributions were more likely at 100 and 150 ms phase offsets (figure 4*b*). Hence, it appeared that when the metronome cues were separated by an offset of around 100 ms or greater, participants did not treat them as a common beat, but rather as independent beats. The bimodal distributions were a result of participants switching their finger taps to be in synchrony with either of the two sources.

We further calculated the mean asynchronies to understand how the timing of participants' movements relative to the onsets of the metronome was affected by the experimental manipulations. We observed changes in mean asynchrony that depended on whether cues best fit a single or dual centred GMM, highlighting the different tapping strategies implemented by participants. For the low phase offsets, mean asynchrony was more positive for the 50 ms phase offset than the 0 ms offset (*F*_1,8_ = 9.31, *p* = 0.016; figure 4*c*), indicating that participants were influenced by both metronome streams and hence integrating the cues. In situations where participants were more likely to show bimodal asynchrony distributions, we observed that one distribution was centred around a negative asynchrony, the other was positive, close to the onset of the second metronome, highlighting the tendency to follow one cue or the other.

### (b) Model fits to the experimental data

The experimental data suggest that human participants apply different strategies under the different experimental conditions: at low phase offsets, data is unimodal with low variance regardless of jitter condition, suggesting integration of the signals takes place. At high phase offsets, data are bimodal and suggest switching behaviour in the use of the two metronomes. This indicates that neither a scenario based on exclusively integrating the timing signals nor one based exclusively on selecting one signal over the other is sufficient to explain the participants' behaviour. Formally, we tested two causal inference models and compared them against models of MI and MS. The first causal inference model (CI) inferred whether the auditory cues originated from a single beat or independent beats using the deviations between the signals caused by both a constant phase-offset and jitter manipulations; the second (CI_PA_) assumed participants adapted to the consistent phase offset between the cues and hence only based their inference on deviations due to the jitter. Using a global search optimization algorithm [25], we fit the four free parameters (see the electronic supplementary material, A, table S2) by minimizing the BIC of each participant's data for each condition and model. Summing the BIC across conditions and averaging for each participant, we were able to compare how well each model explained the data.

**Figure 5.**
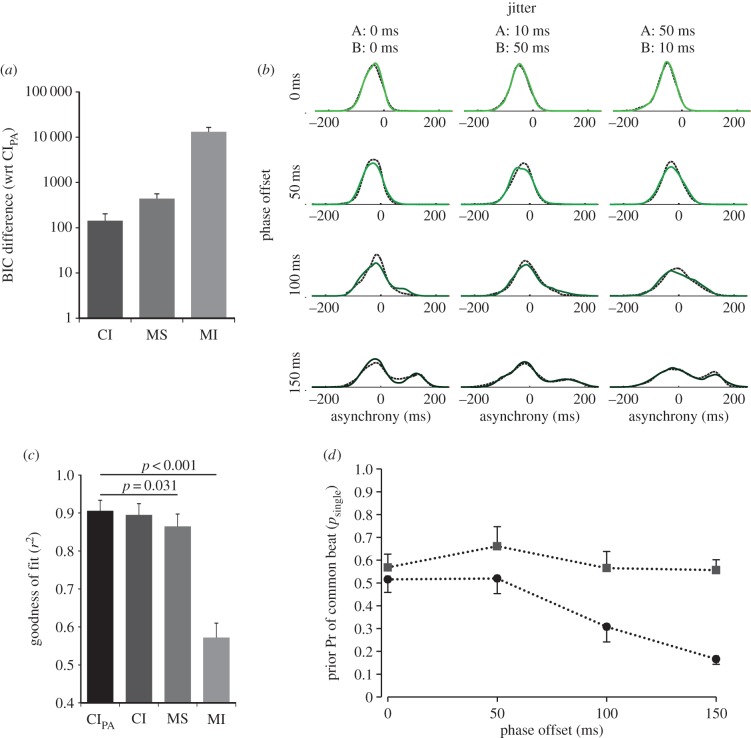
(*a*) Difference in BIC scores for model fits, relative to the causal inference model with phase-offset adaptation (CI_PA_). BIC scores were summed across conditions for each participant. The three alternative models compared were: causal inference without phase adaptation (CI), mandatory separation (MS) of the cues and mandatory integration (MI) of the cues. The difference between BIC scores for each model was calculated and averaged across participants. A positive value indicates that the model is a worse fit to the data compared with CI_PA_. (*b*) Simulated (CI_PA_ model) versus empirical PDFs. The empirical asynchronies (black dashed line) and simulated asynchronies (shaded solid line) were pooled across all participants and converted to PDFs using a Gaussian kernel density estimation (KDE) algorithm. PDFs are shown for each phase-offset condition (rows) and jitter condition (columns). (*c*) Goodness of fit of each model to the experimental data. Goodness of fit was calculated using an index between 0 and 1 by comparing BIC of the data to the model (*L*(*θ*)) relative to: (i) a probability distribution of the data itself (*L*(Max)), and (ii) a random distribution *L*(Rand). Error bars show s.e.m. (*d*) Mean value of the prior probability of a single common beat (*p*_single_) for each offset condition, averaged across participants and jitter conditions. The plot highlights the difference in *p*_single_ values for the CI (squares) versus CI_PA_ (circles) models. Error bars show s.e.m. (Online version in colour.)

We found that, overall, both causal inference models (CI and CI_PA_) outperformed the MI and MS models (figure 5*a*) in terms of the BIC. Specifically, we found that the causal inference models outperformed MI in all conditions and MS in all but one condition (phase offset: 0 ms, jitter: {0, 0 ms}; see table 1). The general goodness of fit measure indicated the simulated data fit well with the experimental data (figure 5*b*) and confirmed differences between the models (*F*_3,24_ = 2.736, *p* < 0.001), with a significantly higher *r^2^* of the CI_PA_ model over the MS and MI models (figure 5*c*).

**Table 1.** Mean difference in BIC between the CI_PA_ model and the mandatory separation (MS) model for each condition. (Positive values indicate the CI_PA_ model is a better fit to the data.)

offset (ms)	jitter (A, B; ms)		
	0,0	10,50	50,10
0	−18.2	3.5	8.3
50	21.4	13.4	18.5
100	48.1	40.7	45.2
150	130.9	104.7	23.0

Importantly, we found that the CI_PA_ model outperformed the CI model in terms of the BIC. This was surprising as the model describes the observer adapting to a fixed phase offset over the course of a trial and discounting this offset when determining whether or not the cues should be integrated. This appears contrary to our empirical results where overall participants' strategies depended on the level of phase offset between the cues. This apparent contradiction can be accounted for by individual differences between participants. In particular, the phase-offset threshold between integration of cues and treating them independently varied across participants, with a minority demonstrating better single-centre (i.e. integration) GMM fits to their distributions even in the 150 ms phase-offset conditions. While the CI_PA_ model introduced an additional fixed parameter in the form of subtracting the phase offset from any estimated deviation between cues (see the electronic supplementary material, A), we noted that this was subsequently modulated by the free parameter *p*_single_ (representing the prior probability of a common single beat). While *p*_single_ remained relatively constant across offsets for the CI model, it dropped as a function of offset for the CI_PA_ model (figure 5*d*). The effect of a reduced *p*_single_ value was to reduce the likelihood of judging a given pair of signals to be a common single beat. Hence, the CI_PA_ model was more able to adapt to the different individual phase-offset thresholds for integration we observed across participants, than the CI model. This resulted in a better fit of the model to each participant's data.

## 4. Discussion

Many simple and skilled actions depend on moving in time with signals that are embedded in complex auditory streams. Often these streams share an underlying rhythm but differ in temporal regularity and phase. Here, we tested how human movement synchronization to two simultaneously presented auditory metronomes was affected by differences in the phase and regularity between the two timing signals. We found that when the phase offset was low, participants showed evidence of integrating the signals, minimizing the variability in the timing of their responses. By contrast, when phase offset was high, responses were more variable and there was alternation in the response cue used for synchronization (*viz*., bimodal distributions of movement asynchronies; figure 4). This behaviour was well captured by a Bayesian causal inference model. The model used four free parameters and was able to explain situations in which participants chose to integrate signals or keep them separate. We applied two causal inference models to the data, one considering phase-offset adaptation (CI_PA_) and one without adaptation (CI). Simulations indicated the causal inference models provided a better account for the experimental data than other models based on integration (MI) or selection (MS) only. The causal inference model incorporating phase-offset adaptation showed a better fit than the causal inference model without phase-offset adaptation. However, the free parameter describing the prior probability of considering the cues to form a single beat was found to be a function of phase offset in the CI_PA_ model. This suggests that the improved fit resulted from this model being more flexible to differences in the phase-offset thresholds at which individual participants switched from integrating cues to treating them independently. Overall, the results suggest that humans exploit a Bayesian inference process to control movement timing in situations where the underlying beat structure of auditory signals needs to be resolved.

Evidence for optimal cue integration for multisensory signals has been demonstrated across a range of tasks in both spatial and temporal contexts [11–13]. Moreover, multisensory cue integration has been shown to result in improved motor performance in a movement timing task [17,29,30]. This improvement was consistent with a maximum-likelihood model of integration based on the reliability of each sensory modality. However, an important step in this process involves deciding whether or not different sensory cues relate to the same environmental event: if not, the signals should be kept separate and not integrated [7–10]. Here, we focused on this process of deciding whether different sensory events relate to a common underlying beat. Our empirical data provided evidence that participants do integrate two auditory signals into a single estimate of a metronome beat, but the probability of integration was a function of both the time offset between the signals and their relative temporal regularity. For instance, when participants tapped to simultaneous beats defined by two metronomes, with one jittered by 10 ms, the other 50 ms, the variability in finger-tap asynchronies remained equal to that when tapping to a single isochronous metronome. This demonstrated that synchronization variability was reduced (relative to the individual signals) by integrating information from the individual (noisy) timing cues. This would not be expected if participants had simply switched between timing cues. Moreover, if participants had simply chosen the more reliable signal, the phase offset between the metronomes would not have been important. By contrast, we found an effect of phase offset, with movement asynchronies for the high phase-offset conditions (100 and 150 ms) producing bimodal distributions of movement timing, indicating that the two streams were treated independently at these high phase offsets. This highlights that a strategy based on integration alone could not account for the participants' behaviour. We suppose two modes of behaviour—integration versus separation, with a causal inference process that decided whether to integrate signals or treat them independently based on their relative reliability and temporal separation. The evidence for causal inference taking place was further corroborated by the single anomalous condition where we found causal inference did not provide the best fit. Namely, when the phase offset was zero and both metronomes were isochronous, we found that a causal inference model did not show a better fit than MS (table 1). This can be explained by the fact that the participants only heard a single metronome cue in this condition (the tones overlap on every beat forming a dyad) and therefore integration could not have taken place. The model was unable to account for this scenario and by integrating the cues described a lower expected variability than was observed, resulting in a poor fit. The predicted poor fit owing to this anomaly provides further evidence that causal inference is taking place in other conditions, where the fit is consistently better than the alternative models.

Exposure to a repeated, consistent asynchrony between multisensory cues has been demonstrated to result in temporal recalibration, such that the point of subjective simultaneity is shifted to compensate for the offset [31]. We considered whether participants would, in a similar way, learn the consistent phase offset between the beats and recalibrate in terms of judging whether those cues defined a common beat or not. We therefore tested two causal inference models: CI_PA_ where the phase offset is accounted for (i.e. not considered) when inferring the causality of the signals, and CI that included the phase offset in determining signal causality. We found that the CI_PA_ model showed a better fit than the CI model. This was surprising given the empirical data showing an effect of phase offset on the distributions. Further examination of the free parameters indicated that *p*_single_ became a function of phase offset for the CI_PA_ model (figure 5*d*). We suggest that these results indicate that participants do not disregard the full phase offset in their inference of a single common beat, but instead account for a proportion of the offset. The CI_PA_ model was more able to account for the differences across participants in the proportion of the phase offset accounted for and hence resulted in a better fit. The results from the model can be considered to show that participants generally underestimate the actual phase offset presented. Under similar circumstances, where repeated exposure to a temporal offset between multisensory cues results in temporal recalibration, it has also been found that an underestimation occurs in the recalibration, explained by a bias in a neural population coding model [32].

Finally, it is interesting to speculate about the cortical circuits underlying the behaviour we observed. There is evidence that different areas of the brain are recruited during beat processing versus duration or interval processing [2,33]: measuring absolute time duration recruits the inferior olive and cerebellum, while if intervals are regular (forming a beat) a striato-thalamo-cortical network is recruited [33]. Here, we added jitter to the metronomes such that the cues presented varied from an isochronous beat (0 ms jitter) through to a highly unpredictable beat (50 ms jitter). Therefore, we may expect a switch from beat-based processing to duration-based activations depending on the level of jitter applied to the metronome. However, by integrating the two cues into a single stream, temporal irregularity is minimized, which is likely to emphasize a beat-based structure. Minimizing the variability and extracting a beat maintains a predictive timing process (rather than reactive) [34], which is what we observed through the typically negative asynchronies to the cue onsets.

In conclusion, when synchronizing actions to auditory streams, people determine whether the cues define a common underlying beat or independent beats through Bayesian inference. As an extension of this work, it would be interesting to investigate the presence of causal inference in real group settings (e.g. a string quartet [5]), using the foundations of the modelling work we have described here.

## Notes

Experimental protocols were approved by the Science, Technology, Engineering and Mathematics Ethical Review Committee at the University of Birmingham.
